# Modeling place field activity with hierarchical slow feature analysis

**DOI:** 10.3389/fncom.2015.00051

**Published:** 2015-05-22

**Authors:** Fabian Schönfeld, Laurenz Wiskott

**Affiliations:** Theory of Neural Systems Group, Institut für Neuroinformatik, Ruhr Universität BochumBochum, Germany

**Keywords:** slow feature analysis, hippocampus, modeling, simulation, place cells

## Abstract

What are the computational laws of hippocampal activity? In this paper we argue for the slowness principle as a fundamental processing paradigm behind hippocampal place cell firing. We present six different studies from the experimental literature, performed with real-life rats, that we replicated in computer simulations. Each of the chosen studies allows rodents to develop stable place fields and then examines a distinct property of the established spatial encoding: adaptation to cue relocation and removal; directional dependent firing in the linear track and open field; and morphing and scaling the environment itself. Simulations are based on a hierarchical Slow Feature Analysis (SFA) network topped by a principal component analysis (ICA) output layer. The slowness principle is shown to account for the main findings of the presented experimental studies. The SFA network generates its responses using raw visual input only, which adds to its biological plausibility but requires experiments performed in light conditions. Future iterations of the model will thus have to incorporate additional information, such as path integration and grid cell activity, in order to be able to also replicate studies that take place during darkness.

## Introduction

Since their initial discovery (O'Keefe and Dostrovsky, 1971) place cells found in the hippocampus were subject to a wide range of both experimental studies as well as theoretical models and ideas. Their main draw is their unusual potential to directly correlate with observable behavior by developing spatially localized fields of activity, the so-called place fields. Place cells also take part in planning (Robitsek et al., 2013) and goal directed behavior (Pfeifer and Foster, 2013). They react to changes in familiar environments and have been shown to adapt their firing rate (local remapping), reposition themselves (global remapping), or become silent, depending on the performed manipulation (Leutgeb et al., 2005a). However, even though their activity has been carefully measured and documented, place field activity has yet to be explained in terms of (neuronal) computation. Theoretical models of place cells do not only have to explain localized firing in the first place, but also need to account for their remapping behavior in order to be able to make compelling predictions for future experiments.

Different modeling approaches can be distinguished by the input they accept and their level of abstraction. While most theoretical work is done in simulation, there are also examples of algorithms implemented on robots. RatSLAM (Milford et al., 2004) employs an architecture that includes competitive attractor networks to successfully solve the SLAM (Simultaneous Localization And Mapping) problem in real time on a robot platform. Though its computational approach significantly differs from ours, RatSLAM operates on a similarly high level of abstraction and also uses raw image data as its input. However, while inspired by hippocampal processing, the goal of RatSLAM is not to explain the emergence of place fields, but to solve the SLAM engineering problem. More biologically motivated approaches usually work on the level of neuronal networks and base their artificial place cell firing on the activity of other cells, such as border cells (Hartley et al., 2000) or grid cells (Solstad et al., 2006). The latter models contribute to a larger debate about whether place field firing is based on grid cell activity (or vice versa) and what their precise nature of interaction is? (Bush et al., 2014).

In this paper we argue that the slowness principle is a fundamental computational principle for place field formation during navigation. We use Slow Feature Analysis (SFA) (Wiskott and Sejnowski, 2002) in a hierarchical network to model parts of the visual system and the hippocampus. The system works with raw visual data as its only input and operates on a systems level rather than a neuronal level of abstraction. This means that despite referring to it as a “network” and “units,” these terms do not denote a set of individual neurons in a clearly defined anatomical region. Instead the model is a self-organizing system implementing the slowness principle to produce place cell firing patterns, in an attempt to explain the computation within the overall hippocampal *system* and not its mechanistic realization by individual neurons. There is also no explicit representation of grid cell activity in the model. While this is a valid approach—place cells have been shown to work properly in the absence of grid cell activity (Hales et al., 2014)—it is not entirely without issue. We will address the matter of grid cells as well as the role of path integration in our framework in the discussion below.

To support our claim of the slowness principle being a central element in hippocampal computation, we replicated six experimental studies, performed on living animals, in simulation. This allows us to compare real-life place cell measurements with the activity produced by our network. Since our model is aimed at the formation of a purely spatial code, we have focused on studies based on (a) random/free exploration and (b) the manipulation of spatial cues in the absence of the animals. These are: two experiments on the effect of cue card manipulations [rotation (Knierim et al., 1995) and removal (Hetherington and Shapiro, 1997)]; two experiments on firing activity depending on the animal's direction [the linear track (McNaughton et al., 1983; Dombeck et al., 2010) and an open field with directed movement (Markus et al., 1995)]; and two experiments on manipulating the borders of a familiar environment [morphing (Leutgeb et al., 2005b; Wills et al., 2005) and scaling (O'Keefe and Burgess, 1996)]. These experiments neither require the animals to understand goal locations nor to memorize specific sequences or timings. The chosen studies include results that we would not necessarily expect from the theory of SFA (Franzius et al., 2007), such as remapping to different firing fields, or stable firing fields in unknown environments. We also discuss the process of measuring place fields over time and demonstrate how different stages of place field development may be explained as mere artifacts of the sampling process.

## Methods

The model used in this work is a hierarchical network that can be mapped to the visual cortex, entorhinal cortex, and the dentate gyrus of the hippocampus proper. It works on a systems level of abstraction and assumes the neural substrate to be sophisticated enough to implement the required computations. In our case these computations are based on one fundamental principle that can be implemented via basic mathematical building blocks.

### The slowness principle

For our work the essential processing paradigm is the slowness principle. It states that more meaningful information within a stream of data changes slower in time than less meaningful information. As an example, consider noisy TV reception: the flickering noise over the actual image varies as quickly as the hardware of the TV is able to produce it. This is much faster than any component of the actual scene you are watching, and thus may be discarded without loss of meaningful information. The slowness principle has first been applied to place field formation by Wyss et al. (2006) where it was used in combination with robotic agents. SFA is an algorithm implementing the slowness principle (Wiskott and Sejnowski, 2002) and has been shown to be applicable to a variety of problem cases (Escalante and Wiskott, 2011) as well as being able to recreate plausible place field firing in both the plain open field (Franzius et al., 2007) and a wide range of other environments (Schönfeld and Wiskott, 2013).

### Slow feature analysis (SFA)

The SFA algorithm is an implementation of the slowness principle. It is an unsupervised learning algorithm that can be trained to extract the most slowly varying components of a continuous stream of input data. Formally: given a multidimensional input signal **x**(*t*) = [**x**_1_(*t*), **x**_2_(*t*), …, **x**_*N*_(*t*)]^*T*^, SFA finds a set of functions *g_1_*(**x**), *g_2_*(**x**), …, *g_*k*_*(**x**) such that each output signal *y*_*i*_(*t*):= *g*_*i*_(**x**(*t*)) varies most slowly over time, i.e.,

(1) $$\Delta \left(y_{i}\right):={\langle {\dot{y}}_{i}^{2}\rangle }_{t}\text{ is minimal}.$$

Note that by this formal definition of slowness the slowest possible signal is a constant value that does not change over time and thus carries no information. To avoid SFA delivering such a constant function as a result, as well as forcing it to not yield the same signal more than once, the following three constraints are defined for the output signals *y_*i*_*(*t*):

(2) $$\text{Zero mean}:{\langle y_{i}\rangle }_{t}\;=0.$$

(3) $$\text{Unit variance}:{\langle y_{i}^{2}\rangle }_{t}\;=1.$$

(4) $$\text{Decorrelation and order}:\;\forall i<j,{\langle y_{i},y_{j}\rangle }_{t}\;=0.$$

As a first step the SFA algorithm applies a quadratic expansion to the set of input signals (cf. Escalante and Wiskott, 2013, for alternative expansions that work well with SFA), and the resulting set is whitened to satisfy constraints (2) and (3). The sought out functions will be linear combinations of the expanded input set. To compute these, the derivative of the data is taken and the covariance matrix thereof computed. The eigenvalue problem of this matrix is solved and the resulting set of eigenvectors denotes the directions of smallest variance within the sphered signal. The elements of each eigenvector now denote the coefficients of the functions *g*_*i*_(**x**) while their respective eigenvalues denote the Δ –values of the extracted slow feature. An alternative version of SFA is described in Berkes and Wiskott (2005); whitening and the standard eigenvalue problem are in this case combined into one step which solves only a single, generalized, eigenvalue problem.

### Hierarchical network

In our experiments SFA is trained with the visual stream of a simulated rat that explores a virtual environment in a random fashion. Single frames of this data consist of 320 by 40 pixel images. In the azimuthal dimension each pixel column represents 1° of the full 320° field of vision (FoV) of the rat (Hughes, 1979), while the 40 pixel range in the vertical dimension represents only a slice of the rat's full vertical field of view. Since wall segments used in the (physical and simulated) experiments do not vary in the vertical dimension, the narrower 40 pixel range still maintains the relevant visual information and helps to reduce the dimensionality of the input. While SFA is able to handle an arbitrarily high number of input dimensions in theory, in practice the algorithm suffers from the curse of dimensionality: SFA requires unreasonable amounts of resources when processing high-dimensional input such as the visual data of our experiments. Specifically, the computational complexity of the SFA algorithm is of order *O*(*NI*^2^ + *I*^3^), with *N* denoting the number of samples and *I* the input dimensionality (Escalante and Wiskott, 2013). We therefore resort to hierarchical processing, which greatly reduces overall computational costs. To construct the hierarchical network from a set of SFA-instancing nodes we use the Modular toolkit for Data Processing (MDP) (Zito et al., 2009). Arrays of SFA nodes form the different layers of the network, and each node processes the data of a predefined receptive field over time. Neighboring fields are set up to overlap each other by half of their size to enable detection of features larger than the size of a single field.

**Figure 1:**
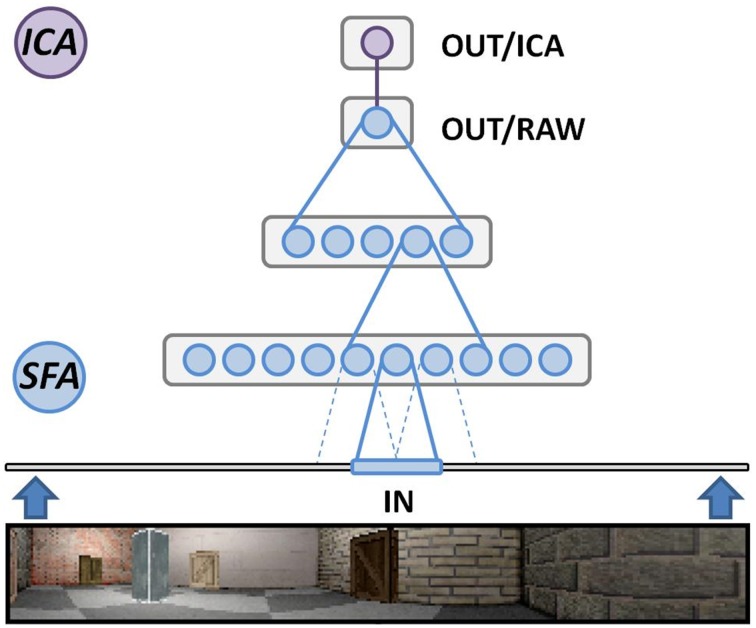
**Overview of our hierarchical Slow Feature Analysis (SFA) network**. SFA nodes in the lowest layer directly work on our software-generated image frames. Each node scans a predetermined receptive field that overlaps 50% with directly adjacent ones. The second layer works on the pre-processed output of the first layer. The third layer consists of a single SFA node integrating the output of all layer-2 SFA nodes. The last layer is one node performing independent component analysis (ICA) to perform sparse coding on the final SFA output.

Figure 1 depicts the structure of the SFA network used in our simulations. The initial three layers are formed from three individual SFA nodes that are each cloned into multiple copies to form their respective layer. The cloned nodes will each perform SFA on their respective input data but share the initial training data. This can be done since we do not expect significant differences in the input statistics at any specific area of the visual field, and further reduces training time of the network. Of the three SFA layers, the initial layer consists of a two dimensional array of 63 by 9 clone nodes featuring a 10 by 8 pixel wide receptive field and 32 output channels each. It works directly on the raw image frames and extracts the most slowly varying signals to relay them to the next layer. Berkes and Wiskott (2005) have shown that the features learned by such a layer resemble those measured in cells of the primary visual cortex. The second layer is defined as an 8 by 2 array of nodes (14 by 6 input channels each) and processes the abstract features provided by the previous layer. The third SFA layer consists of a single SFA instance that integrates all of the features detected over the whole input range by the previous two layers. It can be mapped to the entorhinal cortex and yields the most abstract features extracted from the input data stream. The output signals of this node, however, do not yet resemble clearly localized place field firing patterns. The three initial SFA layers are thus followed by a fourth layer, made from a single MDP node performing sparse coding via independent component analysis (ICA) (Hyvärinen, 1999). This additional step is linked to the dentate gyrus, the hippocampal area one synapse downstream of the entorhinal cortex, where experimental studies have reported sparse firing patterns (Jung and McNaughton, 1993; Chawla et al., 2005).

It is important to note that this network is neither changed nor modified for any specific experiment presented in this text. In fact it is even unknown to the SFA hierarchy that a spatial navigation task is being presented and no heuristics are implemented to make use of any such *a priori* knowledge. The specific parameter values described above are primarily chosen for a division of labor that agrees with the available hardware. The SFA algorithm merely extracts the slowest varying features it is able to detect within a given input stream. If these patterns correspond to the neuronal spiking behavior in real place cells and react qualitatively similar to changes in their input data—i.e., what the rat visually perceives during exploration—we may assume slowness to be an underlying computational principle of a population of such neurons.

While training a hierarchical SFA network instance is fairly time consuming, a fully trained network works in close to real-time and is able to produce its firing patterns without any significant processing requirements. In order to reduce the time it takes to fully train a network we make use of a *generic* training phase. Generic training only trains the first/lowest two SFA layers of our hierarchy and does not use visual data from the actual experiment but rather a set of generic sequences, such as randomly navigating a small set of different mazes featuring a variety of textures and geometric layouts. This enables the lower layers of the network to become familiar with common features of our landscapes, like basic edges, corners, and the horizon (if visible). During the actual experiments only the final two layers are being trained with the data generated during the experiment. Not only does this generic training reduce the overall training time—since only a small remaining part of the network needs to be trained—it is also a much more plausible way to train a biologically inspired model. The visual system does not need to re-learn how to recognize edges with every new room we enter, but instead simply makes use of edge detecting cells that have developed over time.

### Software framework

The training data for our network instances is generated by our freely available Ratlab software framework (Schönfeld and Wiskott, 2013). It allows us to set up any kind of maze layout that is based on a single level (i.e., contains no stairs or ramps). The foraging behavior of the virtual rodent is defined by a number of variables that allow for unguided exploration as well as following user specified waypoints, which is used in two of the six experiments described below. In experimental studies with real animals, random search patterns are generated by tossing food pellets into the mazes. In our model the rat randomly chooses a new direction within its field of view, and employs an additional momentum parameter to smooth its path and avoid unrealistically hectic behavior (Franzius et al., 2007). When instructed to follow a specific path the virtual agent does not move from waypoint A to B in a direct line, but includes a randomized deviation from the optimal path to model a more plausible behavior yielding more realistic input data for our model.

**Figure 2:**
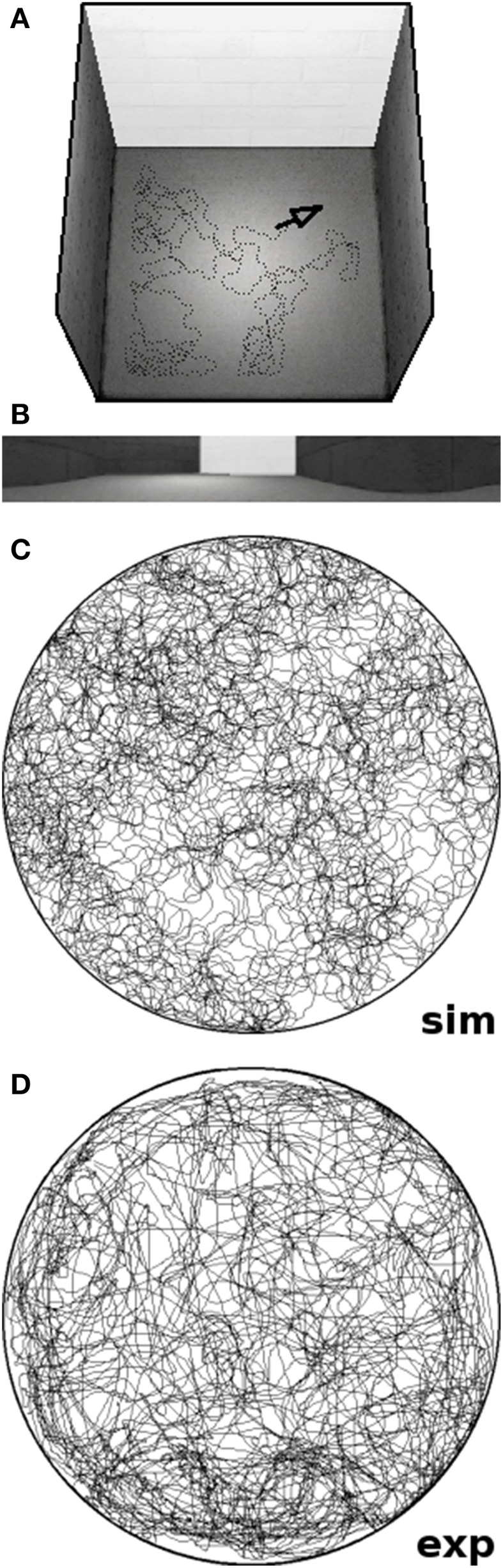
**Screenshots of our software tool**. **(A)** Rendered overview of a simulated square environment featuring a white cue card and the position of the virtual animal marked by a black arrow. **(B)** Exemplary image frame produced by the software; a sequence of such 320 by 40 pixel frames is used to train our network. **(C)** Trajectory of a virtual rat running for 10 simulated minutes within a circular environment. **(D)** Trajectory of a real rat exploring a circular environment of the same radius for 10 min of real time. Note that the virtual agent runs at a constant speed of 20 cm/s and does not slow down or stop as real rats do. Thus, simulated rats tend to cover more ground than their real-life counterparts in the same amount of time.

The overall software framework offers a pipeline of four distinct steps that can be interchanged and concatenated to replicate a wide range of experimental studies (cf. Schönfeld and Wiskott, 2013). Once the setup is defined by the user, the software places the virtual rat within the maze and records the visual stream of the agent while it performs the predefined experiment. The recorded data is then used to train a hierarchical SFA network as described above. The trained network is then sampled over the whole environment and automatically generates the plots presented throughout this work. To replicate the chosen experimental studies we reconstructed the respective environments as closely as the software permits. Maze layouts are matched to scale, similar textures and cue cards are being used, and the background consists of either a closed curtain or an office panorama at a distance (resulting in a realistic parallax). Since the simulated field of view is very narrow in the vertical dimension, however, wall height is usually set to a lower value in order to allow the agent to peek over the walls just like its real life counterpart. Figure 2 shows an example screenshot from our software after concluding a simulation run; it includes a graph of the trajectory of the artificial animal as well an exemplary image frame depicting the view of the agent.

### Experimental protocol

For all simulations presented in this paper the identical base network was used. This network underwent a generic training phase consisting of a set of 100.000 image frames recorded from randomly traversing a variety of virtual environments (a rectangular box, a multiple arm maze, a large square littered with a multitude of cube-shaped obstacles, and a T-maze that also included several such obstacles). For each simulation trial replicating an experimental study, only the uppermost SFA and the top ICA layer were trained with the actual visual data from the experiment considered. The results presented are taken from a single simulation run per experiment, which yields 32 output signals/cell recordings per trial. Of these signals we first selected the ones most similar to the cited experimental results for direct comparison. Where cells depicted a qualitatively different behavior, we selected a range of representative cells from our simulations. This also includes examples of cells with unusual or “misbehaving” firing patterns, if such a behavior was produced by the network. For each result, cell activity is plotted on the commonly used blue-to-red (low to high activity) jet scale. If the selected experimental study has presented its data in a different graphical style we show our results in both their individual and our default format. Note that in contrast to the cited studies, the recorded activity from our model does not correspond to neuronal spikes per time unit. Instead it is a scalar value representing the slow feature as extracted by SFA. This value is not bounded by the algorithm itself, but tends to stay in the reported ranges (see below) unless the network failed to extract a spatial representation. In this case the activity is often erratic and clearly without localized place fields for any output signal component. It is possible to use the SFA output signals to emulate spikes over time (see below), but not only does this process yield no new information, it also results in a reduced resolution when presenting the resulting data.

### Data analysis

To quantify the results produced by our network, we measured both *directional consistency* and *spatial consistency* in terms of the numbers of distinct firing areas for a cell during the different trials. Directional consistency was computed by sampling the full environment with a fixed head direction (N, NE, E, SE, S, SW, W, NW) and computing the average correlation of those fixed-direction plots with the average activity over all views. The data is presented in histograms to show how many cells were firing in a consistent manner independent of the viewing direction. The number of active fields was computed by segmenting firing activity into connected areas of activity above 50% of the cells maximum activity. Activity fields of 25 pixels or less—an area of 5 by 5 cm—were discarded. The data is presented in histograms to show how many cells featured a specific number of firing fields (including zero).

### Simulations

In the following we present seven different experiments, six of which were modeled after previously published studies with real-life animals. While exploring the various environments, the virtual agent was moving at a constant speed of 20 cm/s, without the occasional brake that real-life animals are known to take. Therefore, the virtual rodents in our simulations tend to cover more ground than real animals, whose average moving speed comes down to about 15 cm/s. Though artificial breaks could be added to the simulations, we would not expect this to affect the spatial representation, as long as the breaks happen as randomly as they do with real-life animals. The individual protocols of the actual experiments are as follows.

#### Sampling and development

To examine how place fields are measured over time we set up two simulations: (A) Training of a virtual agent for 8 min in a 60 × 60 cm box built from uniform gray walls with the north wall covered by a white cue card. Walls were high enough to prevent the agent from being able to perceive any surroundings of the maze. Place field activity was sampled over the full environment after 30 s and 1, 2, 4, and 8 min of exploration time. Since SFA cannot be paused and continued we trained five individual networks with successively longer segments of the same overall trajectory. Furthermore, while SFA yields output signals that are ordered by their slowness value, this order is lost when the signals are processed by the uppermost ICA layer of the network. Signals were matched by hand to reconstruct place field development over time. (B) Training of a virtual agent for 8 min in a circular environment also built from uniform gray walls and the northern 90° of arc covered by a white cue card. The radius of the arena was set to 40 cm and the walls again prevented the agent to see the surroundings of the enclosure. Place field activity was sampled while the agent explored the arena *after* completion of the training phase, but only taking into account the space actually visited by the agent after 30 s and 1, 2, 4, and 8 min, respectively. Since this is much closer to measuring place fields in real-life studies, the data from this simulation is also processed as it would in a real-life study: SFA output values are used as firing probabilities to generate artificial neuronal spikes; these are then accumulated in the spatial bins of a regular grid spanning the environment and smoothed over. Note that the need for manual matching described above does not apply in this case, as the training of the network training was not split into different parts.

#### Cue card rotation

This experiment is modeled after Knierim et al. (1995). A circular arena of radius 76 cm is explored for 8 min. Walls are of a uniform gray and a 90° arc of the walls is covered by a white cue card. The arena is surrounded by a black curtain to block out any other visual cues. The network is trained with the visual input stream recorded during the exploration phase. Afterwards the 32 network output signals are sampled over both the original arena as well as a variation where the cue card is rotated by 90° along the wall.

#### Cue card removal

This experiment is modeled after Hetherington and Shapiro (1997). A 83 cm square build from uniform walls with three white cue cards of length 12, 40, and 69 cm attached to the east, west, and south wall, respectively. The apparatus does have a ceiling which for us translates into walls high enough to prevent the agent from looking over them. The virtual agent explored the box for 12 min, after which place fields were sampled multiple times over the whole environment, with different subsets of cue cards removed for each sampling run.

#### Linear track

This experiment is modeled after McNaughton et al. (1983) and Dombeck et al. (2010). A basic linear track of 80 by 12 cm was constructed that bears no distinguishing features but allows the agent to look over its walls to see a surrounding lab environment to orient itself. The virtual agent was instructed to traverse the linear track from one end to the other for 5 min. Traversal control of this path included a noise term to prevent the agent from running along the exact same trajectory during each iteration.

#### Rhombus track

This experiment is modeled after Markus et al. (1995). A circular environment of radius 38 cm was traversed along a rhombus-shaped path for 6.5 min. The path again included random variations to avoid identical visual input for each round along the preset path. Textured walls and a visible laboratory background provided a multitude of available visual cues for orientation. After training, place fields where sampled while following the rhombus-path in both a clockwise and counter-clockwise fashion.

#### Morphed environment

This experiment is modeled after the experiments presented in Leutgeb et al. (2005b) and Wills et al. (2005). The virtual agent explores both a 62 cm square box and a circular arena with a 39 cm radius for 10 min each. Walls are of a uniform color and there are no other distinguishing features within the arena itself. To orient itself the agent is able to look over the walls, where a laboratory background provides distant cues for orientation. After the training phase place fields are sampled over both environments as well as four intermediate stages that represent the enclosure morphing from the square box to the circular arena. The spatial specifications for the in-between stages of the arena were duplicated from Wills et al. (2005).

#### Scaled environment

This experiment is modeled after O'Keefe and Burgess (1996). The simulated rat explores a 120 by 60 cm rectangular box for 10 min. The arena is built from uniform walls that bear no distinguishing marks but are low enough to allow the agent to look over them and orient itself using the global cues of the surrounding laboratory environment. After training, place fields are sampled over the whole arena as well as three additional environments that are constructed in the same way but differ in their dimensions (60 by 120 cm, 60 by 60 cm, and 120 by 120 cm).

## Results

### Sampling and development

**Figure 3:**
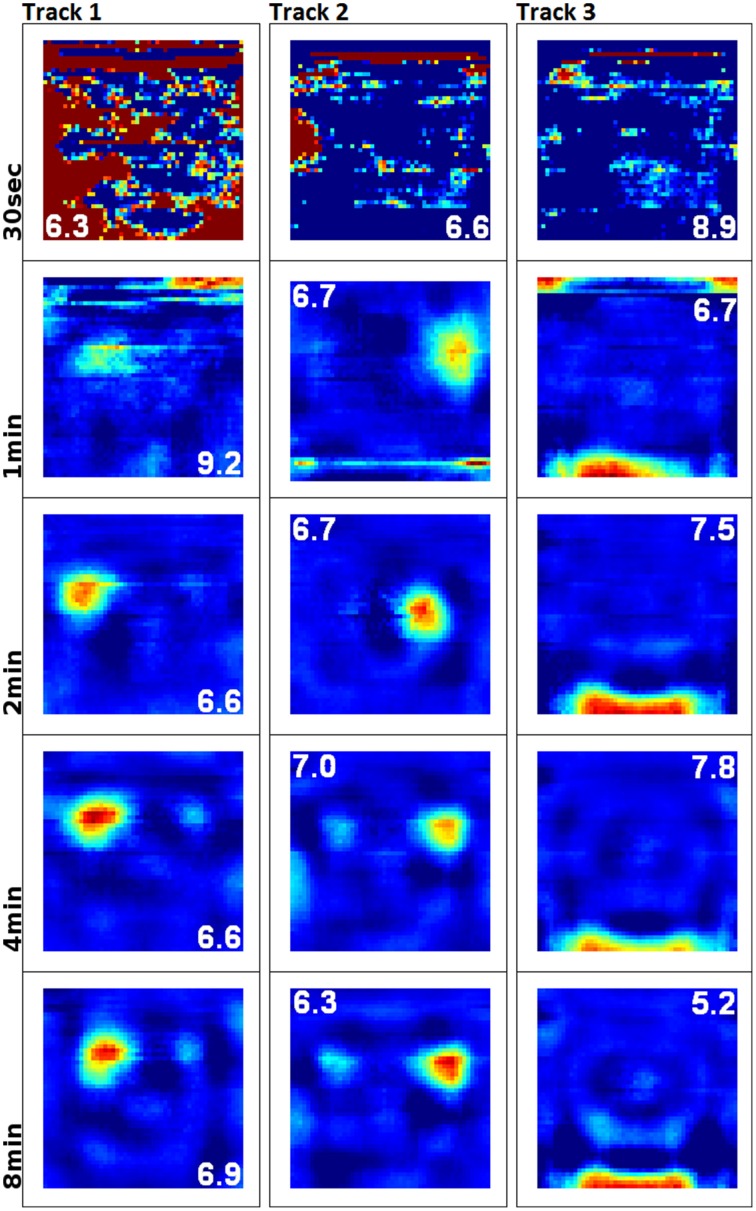
**Development and quality of firing fields as produced by our hierarchical SFA network**. Each row depicts the activity of three cells after exploration of a square environment (white cue card at the north wall) for 30 s and 1, 2, 4, and 8 min of simulation time. Since SFA is not an online algorithm that can be stopped and continued, five individual networks were trained anew along incrementally longer sections of the same overall trajectory. The cells depicted along each column are thus not the same output signals, but rather hand-matched signals produced by the five different SFA networks. This matching was done in order to help compare the quality of place fields over time, and it can be seen that stable and distinct fields develop within 2 min of simulation time.

**Figure 4:**
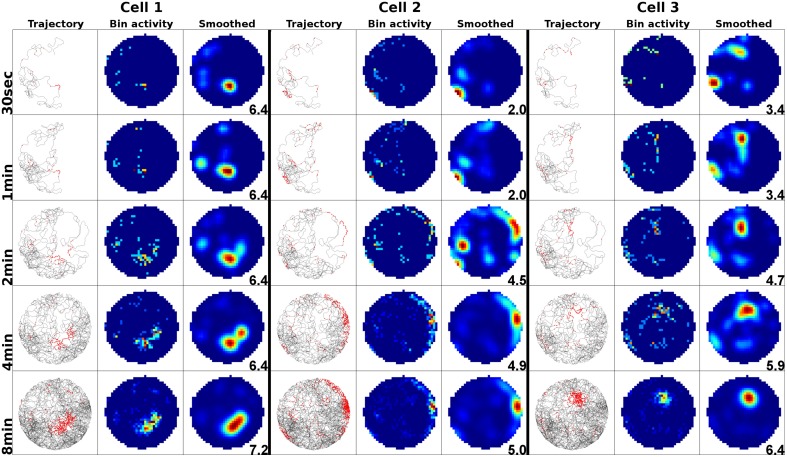
**Sampling of three already established place fields over the course of an 8 min long trajectory**. Despite the place fields being stable from the beginning, they appear to change position and/or split up depending on the coverage of the terrain over time. For each cell three columns are shown; from left to right these are: artificial spikes along the agent's trajectory, based on the activity values produced by our network; accumulated spikes collected in a grid of rectangular bins over the environment; the smoothed bin data as it is commonly presented in experimental papers. The activity was plotted after 30 s and 1, 2, 4, and 8 min of exploration; the number in each row denotes the peak firing activity of the corresponding cell. It can be seen that even though the place fields are stable from the beginning, measurements take time to depict stale firing fields.

**Figure 5:**
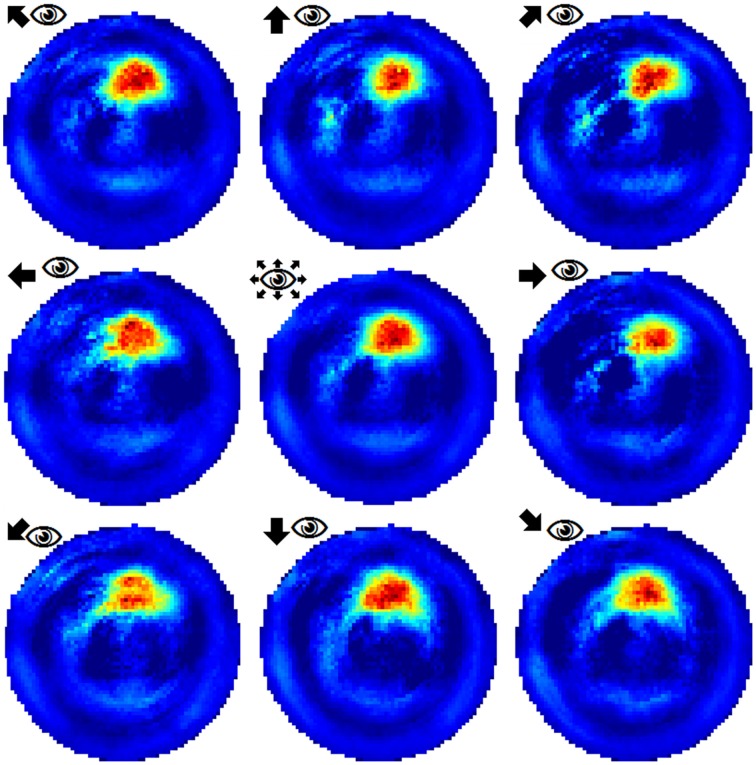
**Example of a place field sampled with the agent looking in eight different directions (N, NE, E, SE, S, SW, W, NW)**. The center image shows the average activity over the surrounding directional plots. Both in experimental studies and our simulations, place fields are found to be largely invariant to the orientation of the (virtual) agent.

To examine the (a) development and (b) sampling issues of place fields we present the results of two different simulations. (a) Figure 3 shows the development of three different place fields after 30 s and 1, 2, 4, and 8 min of exploration time. It can be seen that the hierarchical network produces distinct spatial fields of activity as early as within 2 min of exploration in an unknown environment. (b) Figure 4 shows the emergence of three fully established place fields while the (virtual) animal randomly traverses the environment for 8 min. Network activity is depicted after 30 s and 1, 2, 4, and 8 min of random foraging. Despite the place fields being established from the beginning, Figure 4 shows that it can take up to 8 min before their actual shape becomes clearly visible. It can also be seen that during this time the incomplete sampling leads to the place fields grow (cell 1), split and change position (cell 2), or lose alternative firing locations (cell 3). In addition, Figure 5 shows the firing pattern of a simulated cell as the arena is being traversed while the agent is consistently looking in one of eight different directions. As can be seen, place fields produced by our model fire independently of direction when being trained by random exploration in the open field.

### Cue card rotation

**Figure 6:**
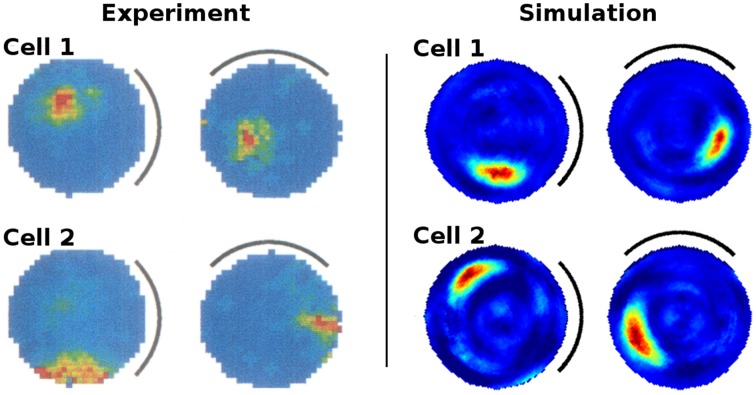
**Rotation of a cue card attached to the wall of an otherwise featureless cylindrical environment**. **Left:** activity of two cell recordings [Reprinted by permission from Society of Neuroscience: Journal of Neuroscience (Knierim et al., 1995)]. **Right:** activity of two cells as produced by our computer simulation. In both experiments and simulations the cue card was rotated by 90° after familiarization and the spatial representation shown to be bound to the single available visual cue.

**Figure 7:**
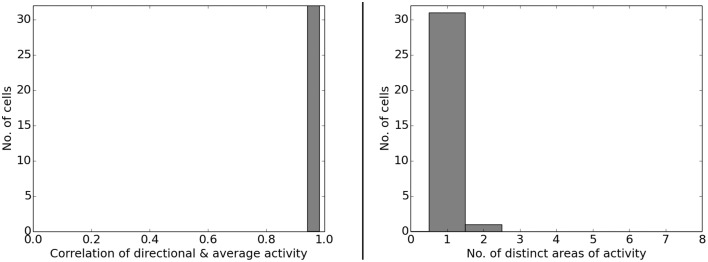
**Left: directional consistency of the artificial cell population during cue rotation**. Right: spatial consistency of the population.

Figure 6 presents the activity of two representative cells before and after the rotation of a white cue card in an environment that lacks any other distinguishing cues. Results from our computer simulations are shown next to the measurements results of real-life animals as reported in Knierim et al. (1995). In both cases the spatial representation can be seen to follow the rotation of the cue card. Directional consistency values for fixed-direction activity over the arena are clustered closely around 1.0, while all cells but one feature a single distinct field of activity (Figure 7). Since the model is based solely on visual information, this result is not unexpected but does confirm a basic ability of the model to generalize: the cue card has to be recognized via edge and color information rather than by memorizing a distinct pixel pattern. Such patterns depend on location and perspective and change when the cue card is rotated.

### Cue card removal

**Figure 8:**
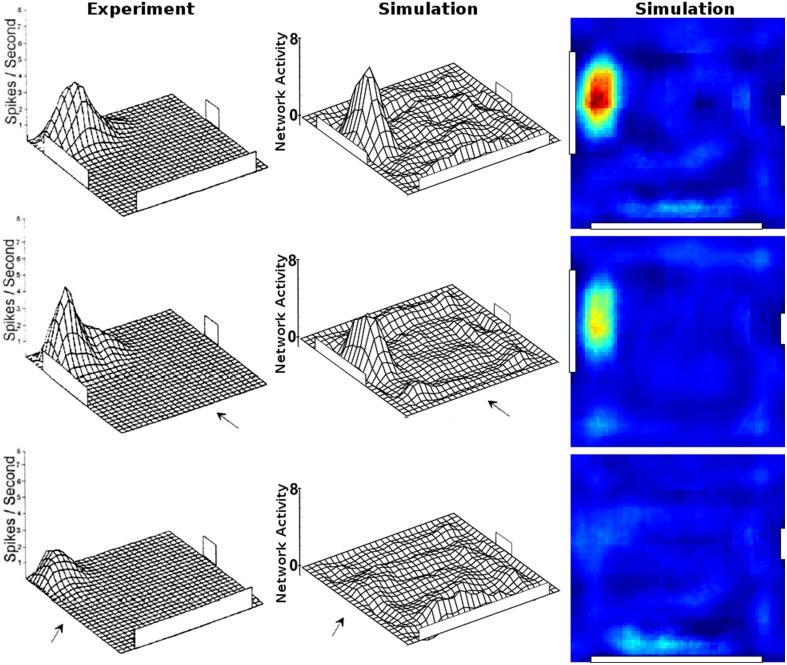
**Changes in place cell firing patterns after the removal of previously learned local cues (three white cue cards fixed to the walls)**. **Left column:** activity from a single cell recording during cue card removal [Reprinted by permission from APA: Behavioral Neuroscience (Hetherington and Shapiro, 1997)]. **Middle column:** activity of the closest matching cell as produced in our simulations. **Right column:** the same cell activity as depicted by the center column formatted with the jet-scale heat map used throughout this work for coherence.

**Figure 9:**
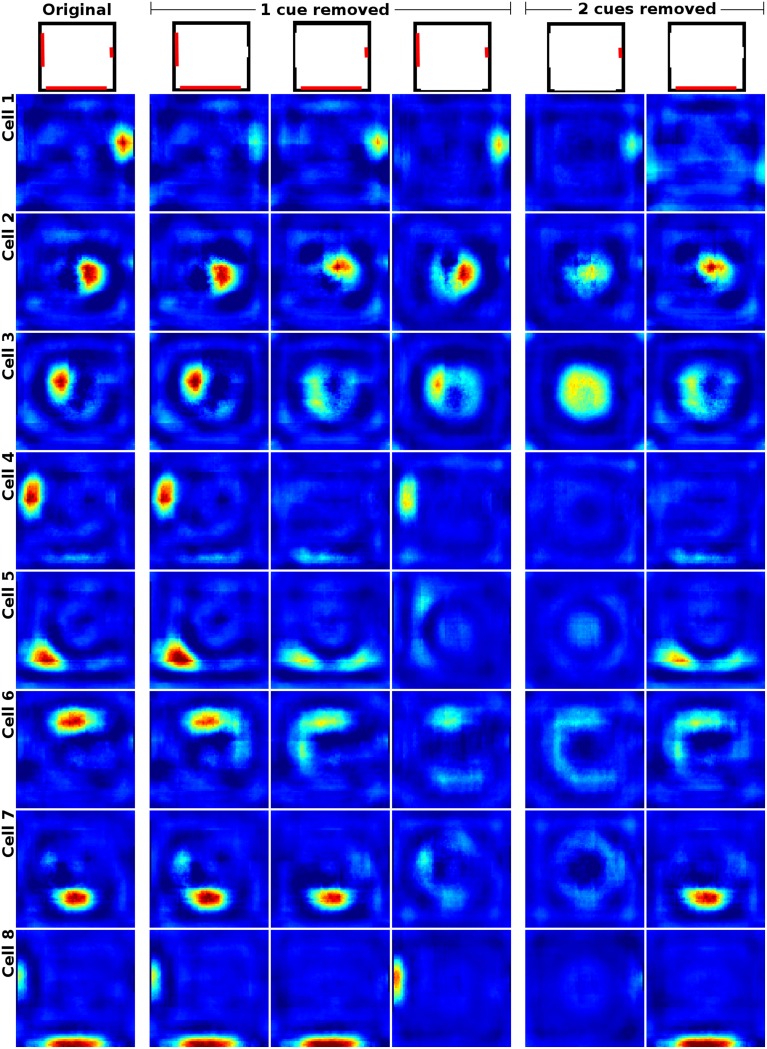
**Cell activity in a square environment with three removable cue cards (red) of different size**. Each row shows the activity of a single cell during the removal of different subsets of the available cues (indicated at the top). Removing the smallest cue affects cell activity the least, with only place fields located close to the missing cue displaying a significant reduction in firing rate. Removal of the medium sized cue card leads to nearby firing fields displaying a distinctive loss in firing activity, while fields positioned further away are impacted considerably less. Removing the largest cue card has the largest overall effect on place field firing. All fields display an explicit reduction in firing activity with fields located near the cue being affected the most. Not all cells behave in the predicted way though and some try to compensate the loss of their associated cue by relocating to a position featuring a similar visual experience (see last row for an example). The rightmost two columns show cell activity with two cue cards removed. In this case most place fields not in direct contact with the single remaining cue card either vanish or develop multiple firing fields which usually are mirrored versions of the original field. This occurs especially in place fields that are located in geometrically distinct but visually indistinguishable locations, such as the corners or the center of the square arena (not shown here).

When removing one of three available cue cards in a square box, Hetherington and Shapiro (1997) report that place field firing patterns change depending on their relation to the removed cue. Fields closer to a missing cue have been observed to display a greater reduction in firing rate than fields near a cue that is still available. Figure 8 shows the firing patterns of a cell in the original experimental study (Hetherington and Shapiro, 1997) and a side by side comparison with the activity of a similarly located place field in our simulation framework. Figure 9 shows the activity of a range of our simulated cells as well as the reaction of the network to the removal of two cue cards at once. As can be seen, our model replicates the findings of Hetherington and Shapiro (1997) and place fields close to removed cue cards tend to be affected more than fields located further away. We also observed cells that reacted in different ways though, such as developing additional, usually symmetric, firing fields; cells that try to compensate the loss of their associated cue by relocating to a position featuring a similar visual experience (see last row for an example); and cells that were specifically anchored to a single cue card. Such fields ignore the absence of cue cards they were not bound to and almost vanish upon removal of their associated cue. Furthermore, removing the smallest cue affects cell activity the least, with only place fields located close to the missing cue displaying a significant reduction in firing rate. Removal of the medium sized cue card leads to nearby firing fields displaying a distinctive loss in firing activity, while fields positioned further away are impacted considerably less. Removing the largest cue card has the largest overall effect on place field firing. All fields display an explicit reduction in firing activity with fields located near the cue being the ones affected the most.

If two cue cards are removed at the same time, fields close to the remaining cue remained intact, though sometimes with a reduction in firing rate especially when the last cue is one of the smaller ones. Place fields at geometrically distinct positions, like the center of the square environment, either remained intact or split into multiple firing fields according to their position. Fields in corners that could no longer be distinguished due to missing cues became active in multiple corners albeit with significantly reduced firing rates. Place cells that were neither anchored to the remaining cue nor placed in geometrically distinct locations usually vanished almost completely.

**Figure 10:**
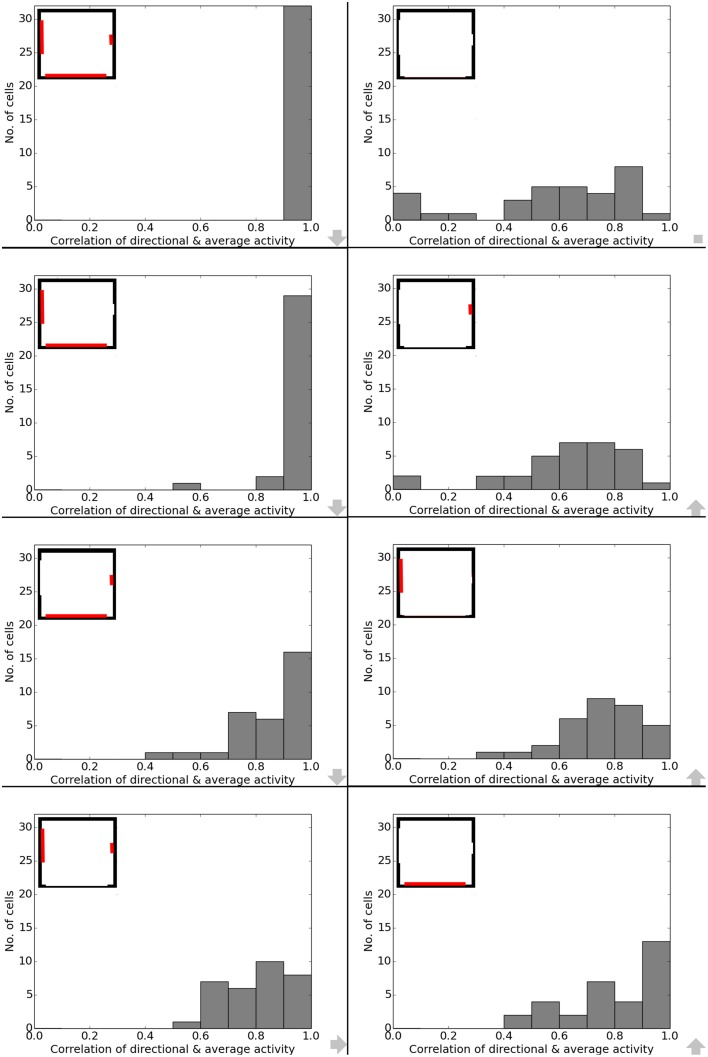
**Directional consistency of the overall cell population during cue removal**. Each plot depicts cell activity in a different version of the familiar environment. Inlets in the top left corner depict which cues (marked red) were still available during sampling of the environment. The order from smallest to largest change in the visual environment is indicated by grew arrows in the bottom right corner of each plot.

**Figure 11:**
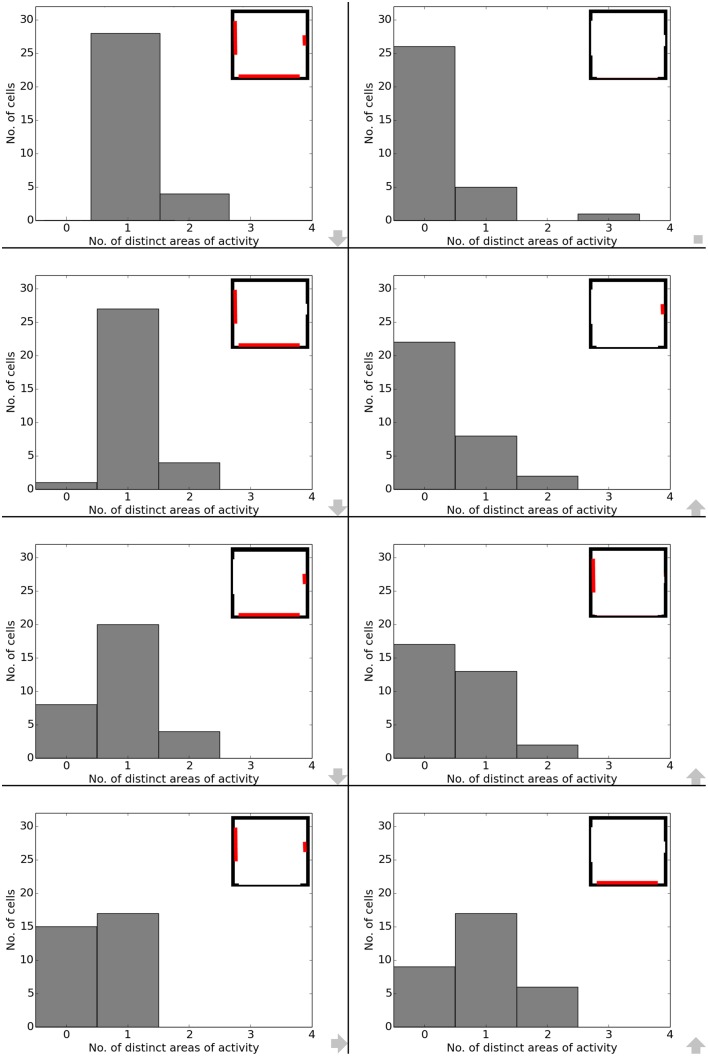
**Spatial consistency of the overall cell population during cue removal**. Each plot depicts cell activity in a different version of the familiar environment holding all three cue cards. Inlets in the top right corner depict which cues (marked red) were still available during sampling of the environment. The order from smallest to largest change in the visual environment is indicated by grew arrows in the bottom right corner of each plot.

Data analysis for all cases shows directional consistency to be higher for environments closer to the initial, familiar state. With the removal of the learned cues, orientation becomes more and correlation values drop accordingly—with larger cues again influencing activity patterns more than smaller cues (Figure 10). The spatial consistency of the cell population can be seen to be influenced in a similar way: cells tend to either lose distinct firing areas or acquire another one, depending on which subset of cues is removed (Figure 11).

**Figure 12:**
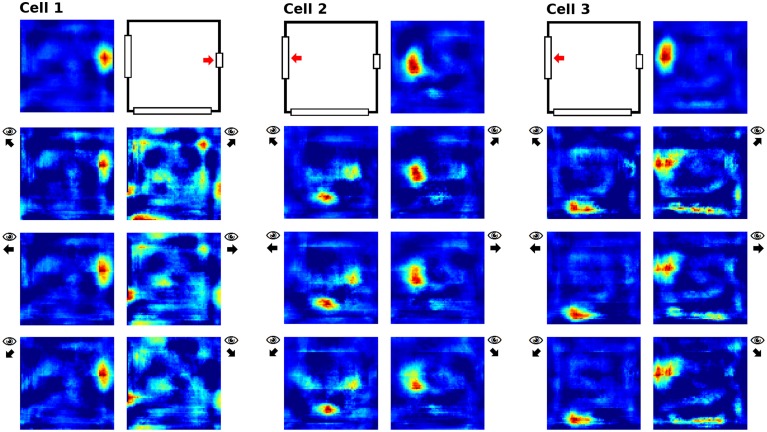
**Place field activity for three cells located in the direct vicinity of cue cards to be removed later**. For each cell, the first row depicts field activity with all cue cards present and indicates the relevant cue card. The lower rows depict cell activity while looking at or away from the relevant cue card; arrows next to these plots indicates the head direction during the sampling process.

Cells in the direct vicinity of a single removed cue also behaved differently depending on whether the virtual rat was facing the cue or not. We assumed this to be the case, since the visual field should not change much when right next to a cue but facing away from it—while activity should be impacted when facing a missing clue at a close distance, since in this case even a small cue card would fill a large section of the visual field. Of the 32 cells recorded, only three cells were located directly next to one of the two smaller cue cards, and their activity in relation to orientation is depicted in Figure 12.

### Linear track

**Figure 13:**
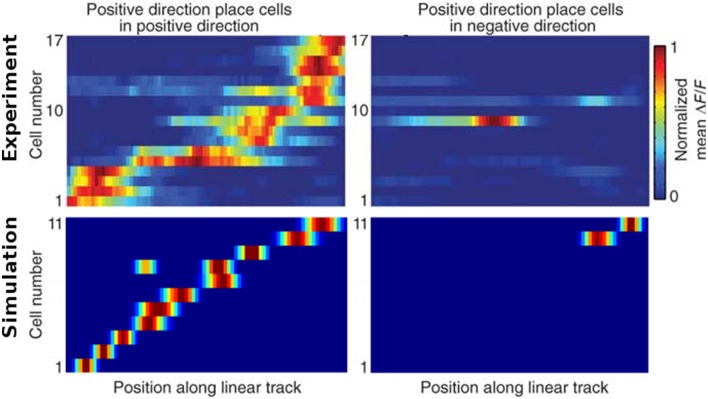
**Firing activity of a range of cells while traversing a narrow linear track in both directions**. **Top row:** cell activity as recorded in animals [Reprinted by permission from Macmillan Publishers Ltd: Nature Neuroscience (Dombeck et al., 2010), copyright 2010]. **Bottom row:** cell activity as produced by our simulations. Left and right images depict activity while the agent moves to the right and left, respectively. In both, the experiment and the simulation, place fields cover the whole track and mostly are only active while the rat/camera faces a particular direction. Note that no simulated place fields are active at the very ends of the linear track, as the virtual animal is told to stop a specified distance before hitting any wall to simulate the extent of a physical body.

**Figure 14:**
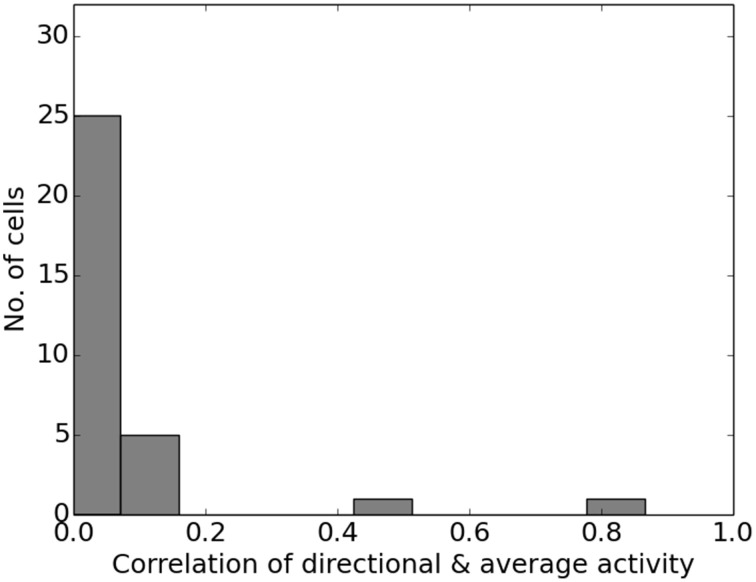
**Directional consistency of the overall cell population within the linear track**.

Figure 13 shows the firing activity of cells while the agent follows a long narrow corridor. In their study, Dombeck et al. (2010) report two distinct properties of the examined fields: (a) they are sensitive to the direction the animal/agent is facing and (b) cover the full length of the linear track. Figure 13 depicts these for both the original recordings and our simulation results. Of the 32 simulated cells, 11 cells did not display any significant firing, 9 cells showed clear direction sensitivity, and 12 cells featured bi-directional activity (two of which featuring two distinct firing locations). Compared to the distribution of cells reported in McNaughton et al. (1983), our results match the percentage of cells that were discarded due to inactivity (34%), while of 25 cells recorded in CA1, McNaughton et al. (1983) reports that “(…) 14 were subjectively classified as highly directional, 6 relatively non-directional, and 5 were ambiguous.” Figure 14 depicts the directional consistency of the overall population, which peeks at zero as expected.

### Rhombus track

**Figure 15:**
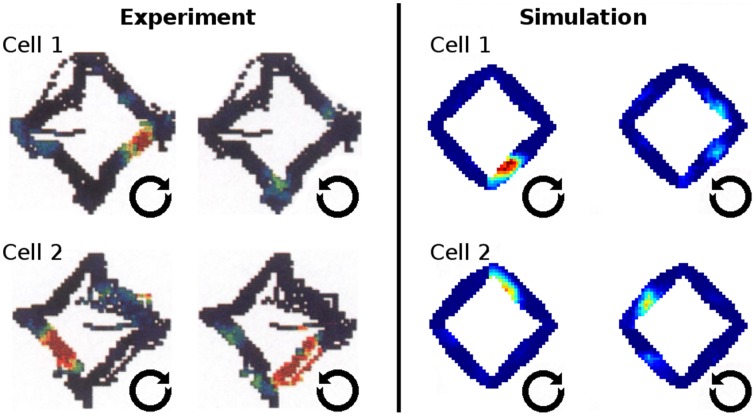
**Firing activity in the open field after animals were trained to follow a rhombus-shaped path in both a clockwise and a counter-clockwise fashion**. **Left:** firing patterns of two cells as the open field is traversed by animals along the trained path in two orientations [Reprinted by permission from Society of Neuroscience: Journal of Neuroscience (Markus et al., 1995)]. **Right:** firing patterns of two cells as produced by our simulations. Both cases depict direction sensitive activity despite the (virtual) agents navigating in an open field where firing is usually reported to be invariant to orientation.

**Figure 16:**
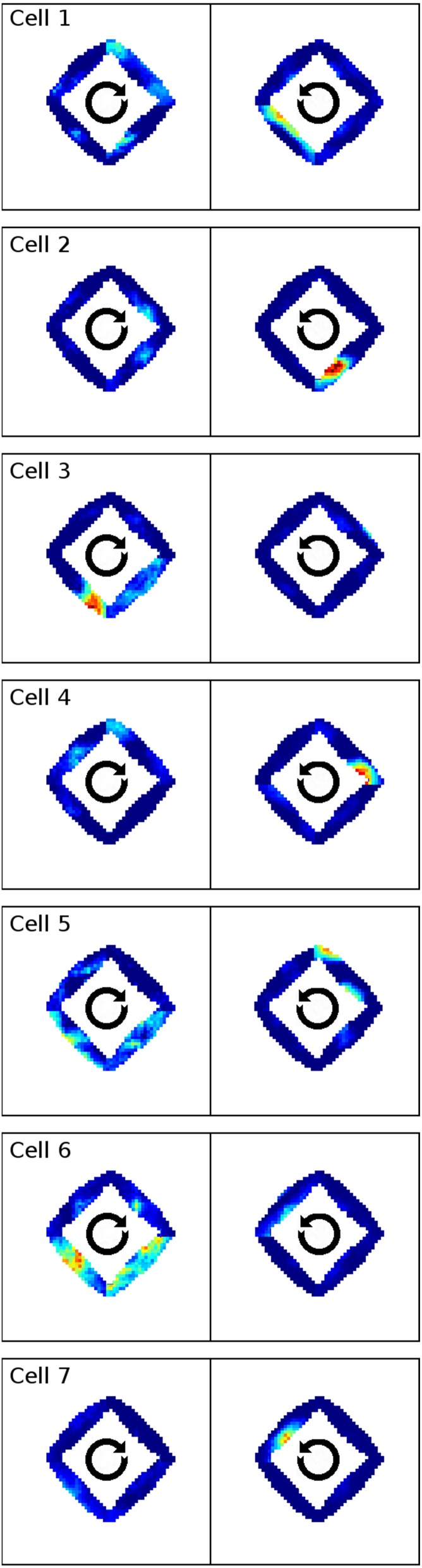
**A representative sample of firing activity in the rhombus-track experiment as produced by our simulations**. Each row depicts the firing activity of one cell during both clockwise and counter-clockwise traversal of the open field along the pre-trained rhombus-shaped path. All cells can be seen to be sensitive to a specific orientation and lose their distinct firing fields when traversed in the other direction.

**Figure 17:**
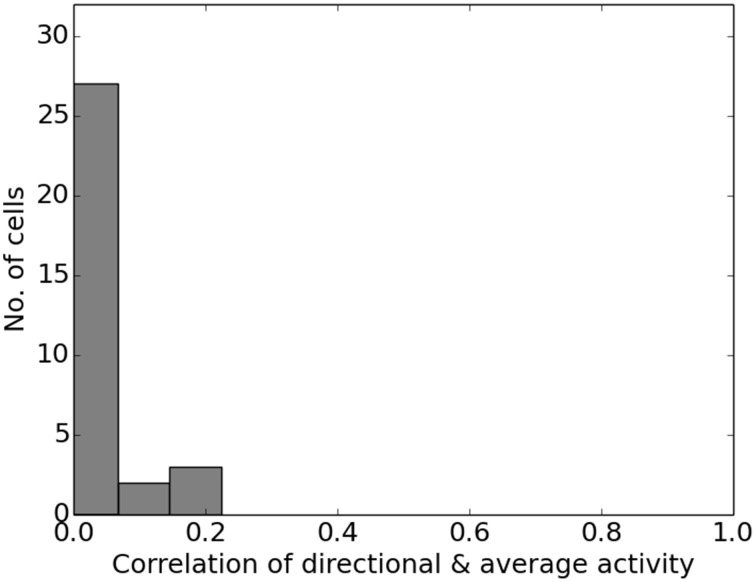
**Directional consistency of the overall cell population within the area covered by the virtual agent during training in the rhombus track experiment**.

In Markus et al. (1995) animals were trained to follow a rhombus shaped path within an open environment. Measuring the animals' place code revealed directional dependent firing. Figure 15 shows one such cell as reported in Markus et al. (1995) in a side by side comparison with a hand-picked cell from our simulation. Figure 16 shows a number of representative cells from our simulation results and their firing activity when the animal follows its trained path in a clockwise and counter-clockwise fashion. Of the 32 cells measured in our simulation, 16 cells showed clear place fields in one direction and little to no activity in the other; 8 cells showed clear fields in one direction and significant but unstructured activity in the other; while the remaining 8 cells showed either unstructured activity in both directions or stayed silent. Figure 17 shows the directional consistency of the cell population, which for this second direction sensitive setup again peeks at zero.

### Morphed environment

**Figure 18:**
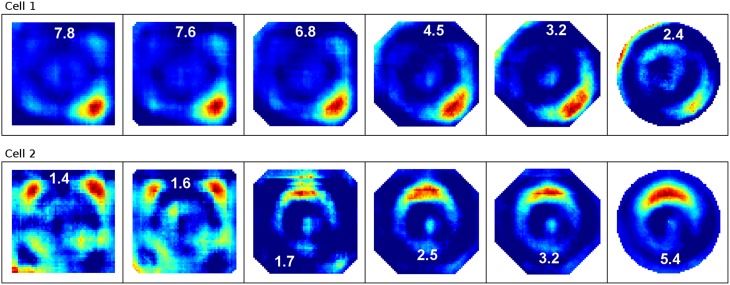
**Firing activity of cells that were trained in both a rectangular and a circular environment: each of the two rows depicts the firing activity of one cell as the environment morphs from a rectangular layout to a circular one**. Firing rates decrease steadily as the environment morphs into the shape a cell is not anchored to while the network attempts to keep the fields in the same positions.

**Figure 19:**
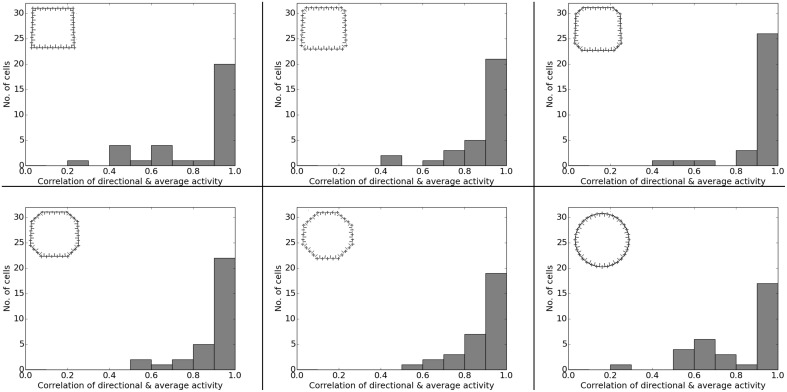
**Directional consistency of the overall cell population in the different stages of the environment-morph experiment**. Each plot depicts a different stage as indicated by the respective diagrams in the top left corner of each plot.

**Figure 20:**
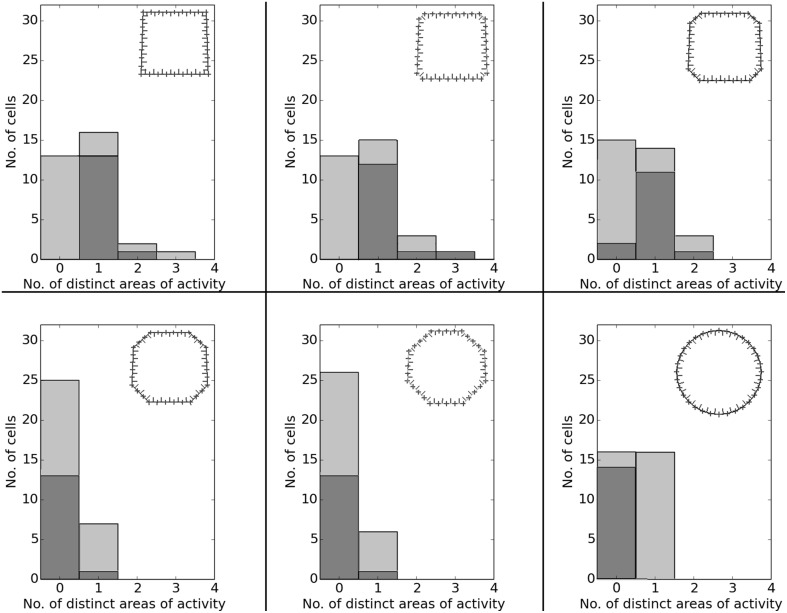
**Spatial consistency of the overall cell population in the different stages of the environment-morph experiment**. Each plot depicts a different stage as indicated by the respective diagrams in the top right corner of each plot. Cells with preferred firing in the rectangular area are dark gray; cells with preferred firing in the circular arena are light gray.

Both Wills et al. (2005) and Leutgeb et al. (2005b) studied the effects of morphing the environment from a familiar square box to an also familiar circular arena, with the in-between morph states being unfamiliar to the animals. Figure 18 shows the activity of two representative cells over the whole arena during the different morph stages. In our simulations, cells usually have one distinct firing field in either the box or the circular arena. Field activity stays in the same location (as far as the geometry of the maze layout permits) and gradually fades out as the environment morphs from a rectangular configuration into a circular one and vice versa. Figure 19 shows how this forms the statistics of the overall population: in both the square box and circular arena around half of the cells show directional consistency. It can also be seen how this value drops for all cells during the in-between morph stages of the arena. Similarly, in both the square box and the circular arena around half of the population shows a single firing field, while the other half remains silent (Figure 20).

### Stretched environment

**Figure 21:**
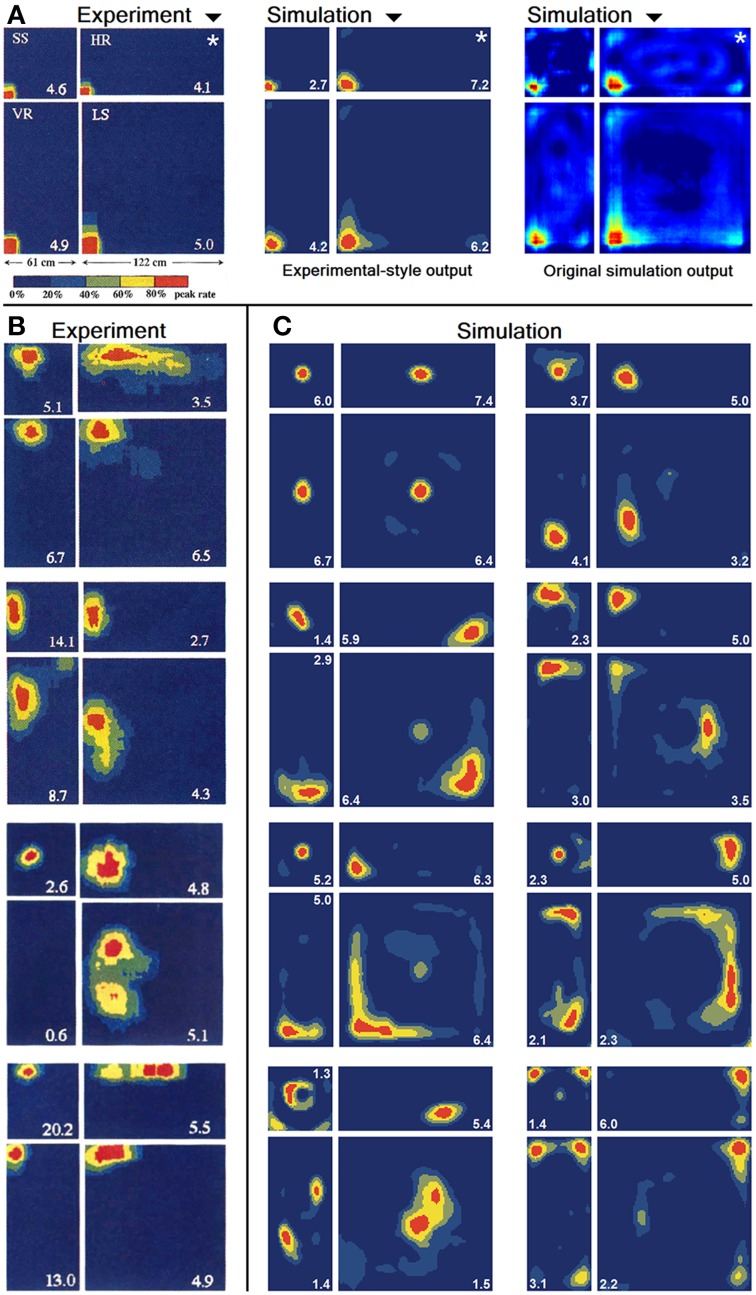
**Firing activity of a variety of cells within the original environment they were trained in (marked by a white star in A) and differently scaled versions of it**. **(A)** Firing of a single cell as reported in O'Keefe and Burgess (1996) (left) and a handpicked, similarly behaving cell as produced by our computer simulations (middle). The same virtual cell is depicted to the right in the original format used by our software for comparison reasons. **(B)** Firing activity of four different cells recorded from rats [Reprinted by permission from Macmillan Publishers Ltd: Nature (O'Keefe and Burgess, 1996), copyright 1996]. **(C)** Firing activity of eight different cells as produced by our simulations.

**Figure 22:**
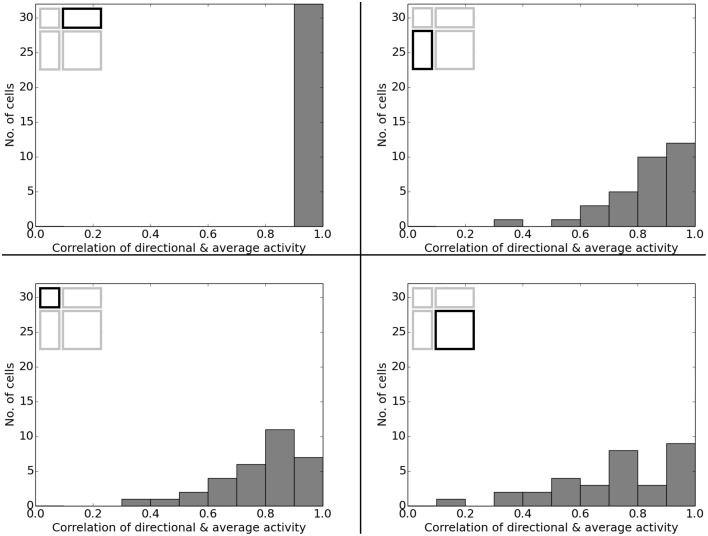
**Directional consistency of the overall cell population in the various scaled versions of the familiar 120 × 60 cm arena**. Each plot depicts a differently stretched version as indicated by the corresponding diagrams in the top left corner of each plot.

**Figure 23:**
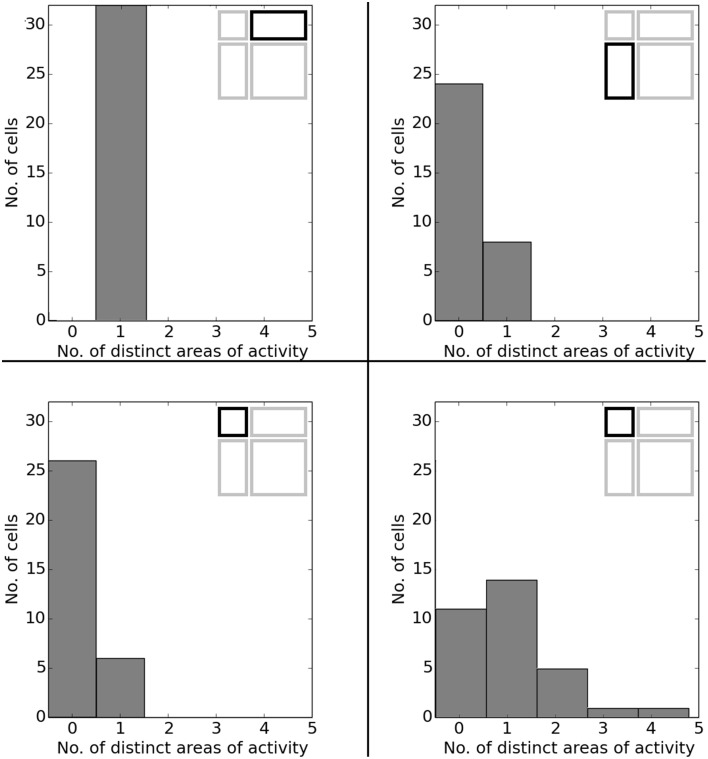
**Spatial consistency of the overall cell population in the various scaled versions of the familiar 120 × 60 cm arena**. Each plot depicts a differently stretched version as indicated by the corresponding diagrams in the top right corner of each plot.

In O'Keefe and Burgess (1996) animals were trained to become familiar with a rectangular environment and then have their place cells being recorded from while exploring a number of differently scaled versions of the original arena. In their study, they reported a range of observations: fields were seen to stay at fixed distances from certain sections of the walls, stay at relative positions within the overall maze, and getting pulled apart when the maze was being stretched out. Figure 21 shows a number of results reported in O'Keefe and Burgess (1996) as well as a collection of selected cell activity as computed by our model, including one cell with a striking resemblance to the one reported in O'Keefe and Burgess (1996). The images shown were chosen to demonstrate the observed range of behavior, including the classes reported in O'Keefe and Burgess (1996). All 32 cells simulated in our model showed clear spatial activity in the original 120 × 60 cm setting as well as in the morphed versions—i.e., none of the cells broke down into an incoherent excess of activity, which usually happens if the model is unable to properly process its input. For almost all of the cells the activity in the morphed boxes can be labeled as either belonging to one of the categories reported in O'Keefe and Burgess (1996) or to one of the additional classes observed in our simulations: fields that rotated to stick with the same relative position to distinct landmarks (such as corners, for example); fields splitting into two, usually mirrored fields; and fields that relocated (often to the center of the new environment and usually observed in the square variants). While the firing patterns stayed coherent in almost all 32 cases, two units displayed an overall loss of localized firing, with different “mutations” of the original field in each of the stretch variants. Figures 22, 23 depict the statistics of this behavior for the overall population. While directional consistency is close to 1.0 for the familiar 120 × 60 cm environment, this value drops for most cells in the scaled versions of the arena, with the large 120 × 120 environment being the most confusing to the network. The number of firing fields behaves in a similar fashion, and cells can be seen to fall below 50% of their familiar-setting peak activity in the unfamiliar settings.

## Discussion

In this section we put our results in context and discuss our hypothesis of the slowness principle being a fundamental building block in hippocampal processing. Afterwards we look at the shortcomings of the model in its current state, and talk about the role of path integration and grid cells.

### Place field development

In Figure 3 we have shown that our model produces stable firing fields after only 2 min of exploration. In the real-life experiments this process is usually reported to take significantly longer, generally around 8 to 12 min. In order to reconcile this difference in development time, we ask: assuming animals would be able to create a stable spatial representation within 2 min, would we be able to measure this? Figure 4 shows the answer to this question to be no. We can see that even with stable firing fields from the start, it is very difficult to verify their position and size until the agent has sufficiently sampled the environment. In a suitably small and plain arena, as they are commonly used in real-life experiments, we show that this process takes about 8 min—note, however, that due to the higher average speed of our virtual agents, real-life rats might need up to 25% more time to fully sample an environment. Additionally, we observe the same phenomena that are reported when the “development” of real-life place fields is being tracked: fields seem to shift position, split, and merge. As can be seen, we do not need to look for a complicated mechanism that is responsible for this behavior, since we can explain it as a logical consequence of the sampling process.

### Place fields and cue control

It is well-established that prominent visual cues help to anchor the spatial representation of the environment, and this is also the case in our simulations (Figure 6). Removal of one or more cues leads to remapping of cells, as demonstrated in real-life animals by Hetherington and Shapiro (1997) and our simulations (Figure 9). Hetherington and Shapiro (1997) suggest that the remapping is based on a distance measure: “(…) evidence that [Place Cells] encode information about distances between the organism and environmental stimuli.” While it would be difficult to discard this hypothesis based on our results alone, it is possible to propose an alternative that does not require the addition of a distance metric in order to explain the observed results: assuming all cues are of the same size, then a distant cue takes up less space within the field of view (FoV) than a closer cue. Therefore, if a place field is established right next to a prominent cue, this cue will almost always take up a significant amount of the very wide FoV of the rat. It follows that the removal of a closer cue changes the visual input at this location more than the removal of a cue that is located further away. A network that associates visual input with location will thus significantly reduce the firing if a cue close to a firing field is removed. This explanation of the observed remapping is obviously still based on distance, but now the notion of distance is something to be concluded from the difference in firing upon cue removal instead of being required to compute that difference in the first place. This hypothesis has two implications: (a) when directly looking away from a nearby cue, place field activity should be impacted a lot less if the cue is removed, since the cue takes up less, if any, space within the FoV in either case; (b) a larger cue should impact place field activity even if it not close, since it takes up significant space in the FoV in both cases. The former can be seen in Figure 12, the latter in Figure 9. In a more abstract sense, this suggests that the deciding factor to explain the described observations is to be found in the input statistics, and not an additional mechanism like distance measurement.

### Directional dependency

Place cell activity that depends on the direction the animal is facing has been known for some time. The linear track has been proven a reliable method to investigate this behavior, as shown in McNaughton et al. (1983) where it was observed in the arms of a multiple-arms apparatus, or as reported in Dombeck et al. (2010) where mice were held in position on top of an air-cushioned ball and ran along a virtual track projected into their field of view. Our own simulation setup took cues from both of these studies, and in all three variations we can see place cell activity that depends on the head direction of the animal, as shown in Figure 13. Such directional specificity is usually not observed in rats that randomly explore an open arena. Presumably this is because, over time, every location is traversed from different angles and thus overlapping views can be associated with the same location—and with the rat's wide field of view, most visual impressions at the same location can be expected to overlap. It should thus not be the layout of an environment that leads to place cells being sensitive to the animal's orientation but rather the agent's movement pattern and thus the statistics of the visual input. This idea is supported by the study by Markus et al. (1995) where animals were trained to follow a rhombus-shaped path on the floor of a cylindrical platform without walls. Place field activity was then measured while the animals followed this path in both a clockwise and counterclockwise orientation. Despite no further geometry obstructing the animal's view, clear directional dependent firing was shown both in their original study with real-life animals and our virtual version of it (Figure 16).

### Remapping in morphing environments

Re-arranging a familiar environment is a different class of spatial modification compared to the modification of cues within an otherwise unchanged arena. Two central questions may be addressed in this fashion: (a) At what point does a familiar environment become unfamiliar enough to be judged a different environment and thus warrant global remapping? (b) How do cells adapt to subtle but successively increasing changes of a familiar environment; and does it happen gradually or suddenly? The study of O'Keefe and Burgess (1996) examines the first of these questions and reports remapping of place fields that seems to follow clear patterns: place fields stick with both relative and absolute spatial features such as corners or stretching along walls, and relocate appropriately in scaled versions of the original environment. Our simulation results behave in a similar way (Figure 21) and support the hypothesis that, despite clearly being recognized as not the same, scaled environments are not treated as entirely different. The spatial representation upheld by the animals' place cells can thus be adapted and re-used and does not have to be acquired anew.

To address the second question, both Wills et al. (2005) and Leutgeb et al. (2005b) trained animals in two distinct arenas—one square and one circular—before measuring their place cell activity in a number of intermediate stages. However, while Wills et al. (2005) report a sudden shift in the place cell's spatial representation from one of the intermediate stages to the next, Leutgeb et al. (2005b) observe a gradual shift in firing activity from the first environment to the last. Despite a thorough discussion, (Leutgeb et al., 2005b), as the later of the two studies, is unable to point to a single distinguishing feature that differs from one experiment to the other and would help to explain this divergence of results. Therefore, it is difficult to judge whether our own results match the “correct” real-life recordings. As can be seen in Figure 18, firing activity in our network would seem to suggest that a gradually shifting representation is supported by our theoretical work. However, the Wills et al. (2005) study originally set out to find evidence for pattern separation in the place cells of area CA3 due to their recurrent connections—which would predict the sudden shift in activity that they do observe. Because of this, comparing the output of our model to their measurements directly is problematic, since our network lacks any kind of recurrent connectivity. The gradual shift observed in our simulations would lead to a sudden shift if it was taken as the input to a second network featuring recurrent connections. As long as activity stays above a certain threshold, recurrent connections would reinforce firing activity and thus allow the network to stay in this particular attractor state. Dropping below such a threshold would, by the same reasoning, force the network into a different attractor state, where the formerly active cells now become silent.

### Grid cells

In this paper we have shown how a computational model can be based on visual input alone and produce biologically plausible place field activity in a variety of experiments. Our model does not include a path integration system, however, nor does it take grid cell activity into account. This is an unusual approach in a field where a lot of work is dedicated to investigating the issue of how exactly grid cells interact with place cells. It is widely assumed (Burgess and O'Keefe, 2011) that these two systems, one based on path integration and one based on visual cues, work together to facilitate the full navigation abilities of rodents. We consider the interplay between these two systems as supportive rather than strictly required. The primary benefit of these systems cooperating is not to enable each other in the first place, but to compensate for their corresponding weaknesses. Navigating with visual cues alone will fail in darkness, for example, while depending on path integration alone will confuse the agent due to the accumulating error in the motor system. Working spatial representations without grid cell input are supported by Hales et al. (2014), who have shown that place cells can operate in the absence of functional grid cells, and Brandon et al. (2014), who have shown that new place field representations are developed and retained in unfamiliar environments during the inactivation of grid cell activity. In order to replicate experiments that include stages in darkness, such as (Quirk et al., 1990), or the so-called “teleportation trials” (Jezek et al., 2011), though, path integration and grid cell activity will have to be part of the modeling approach.

## Conclusion

In this paper we have demonstrated that a hierarchical SFA/ICA model is able to establish, without any prior information, a stable spatial representation akin to place cells found in real-life rodents. We found good agreement of our simulations with results from six different experimental studies that manipulated the environment in different ways. Since our model is exclusively based on the hierarchical application of the slowness principle—plus one linear sparse coding transformation—this provides evidence for slowness to be a likely candidate for basic neuronal processing in the mammalian brain.

### Conflict of interest statement

The authors declare that the research was conducted in the absence of any commercial or financial relationships that could be construed as a potential conflict of interest.
